# Measured and Predicted Resting Energy Expenditure in Malnourished Older Hospitalized Patients: A Cross-Sectional and Longitudinal Comparison

**DOI:** 10.3390/nu12082240

**Published:** 2020-07-27

**Authors:** Maryam Pourhassan, Diana Daubert, Rainer Wirth

**Affiliations:** Department of Geriatric Medicine, Marien Hospital Herne, Ruhr-Universität Bochum, 44801 Bochum, Germany; Diana.Daubert@elisabethgruppe.de (D.D.); Rainer.Wirth@elisabethgruppe.de (R.W.)

**Keywords:** resting energy expenditure, indirect calorimetry, Harris–Benedict formula, malnutrition

## Abstract

A number of equations have been proposed to predict resting energy expenditure (REE). The role of nutritional status in the accuracy and validity of the REE predicted in older patients has been paid less attention. We aimed to compare REE measured by indirect calorimetry (IC) and REE predicted by the Harris–Benedict formula in malnourished older hospitalized patients. Twenty-three malnourished older patients (age range 67–93 years, 65% women) participated in this prospective longitudinal observational study. Malnutrition was defined as Mini Nutritional Assessment Long Form (MNA-SF) score of less than 17. REE was measured (REE_measured_) and predicted (REE_predicted_) on admission and at discharge. REE_predicted_ within ±10% of the REE_measured_ was considered as accuracy. Nutritional support was provided to all malnourished patients during hospitalization. All patients were malnourished with a median MNA-LF score of 14. REE_measured_ and REE_predicted_ increased significantly during 2-week nutritional therapy (+212.6 kcal and +19.5 kcal, respectively). Mean REE_predicted_ (1190.4 kcal) was significantly higher than REE_measured_ (967.5 kcal) on admission (*p* < 0.001). This difference disappeared at discharge (*p* = 0.713). The average REE_predicted_ exceeded the REE_measured_ on admission and at discharge by 29% and 11%, respectively. The magnitude of difference between REE_measured_ and REE_predicted_ increased along with the degree of malnutrition (*r* = 0.42, *p* = 0.042) as deviations ranged from −582 to +310 kcal/day in severe to mildly malnourished patients, respectively. REE_predicted_ by the Harris–Benedict formula is not accurate in malnourished older hospitalized patients. REE measured by IC is considered precise, but it may not represent the true energy requirements to recover from malnutrition. Therefore, the effect of malnutrition on measured REE must be taken into account when estimating energy needs in these patients.

## 1. Introduction

It is well known that aging is associated with a reduction in resting energy expenditure (REE) mainly due to loss of fat free mass (FFM). However, after adjustment for body composition, older persons have significantly lower REE compared to younger adults [1,2]. This decline in metabolic rate results in lower energy intake and is associated with nutritional deficits in older individuals [3]. Outside of age and body composition-related changes in REE, other factors, such as malnutrition, may influence REE in older individuals. Malnourished older persons may have a lower REE, due to metabolic adaptation to low energy intake. Consequently, measured REE may decrease because of malnutrition, and may not represent a patient’s true energy requirements.

REE can be measured by indirect calorimetry (IC), which is a noninvasive technique and considered as the gold standard in hospitalized patients [4]. By using IC through measuring oxygen consumption and carbon dioxide production, changes in energy metabolism can be detected. In this manner, REE data can be used for individualized nutritional therapy, which helps patients receiving the required amount of energy at each phase of treatment. However, the use of IC is limited in daily practice, due to the high cost of equipment and the time-consuming measurement.

Alternatively, a wide variety of predictive equations is used to estimate patients’ REE. However, most of these equations are based on healthy not hospitalized patients and the majority was not specifically developed for geriatric patients. In addition, previous studies have demonstrated a major discrepancy (i.e., overestimation or underestimation) between measured REE by IC and the values calculated with these formulas in older hospitalized patients [5,6,7]. Therefore, it can be hypothesized that most of these predictive equations are not sufficiently accurate to estimate REE in older patients, and there is no consensus regarding which equation should be used in this population. However, the Harris–Benedict formula [8], which is based on anthropometric variables is the best known and precise equation to calculate REE [4]. Thus, in the present study, we aimed to compare REE measured by IC and REE predicted by the Harris–Benedict formula among 23 malnourished older hospitalized patients.

## 2. Materials and Methods

The study population consisted of 23 malnourished older participants (15 women and 8 men) with an age range between 67 and 93 years who consecutively hospitalized between September 2019–January 2020 at a geriatric acute care unit, at the university hospital, Marien Hospital Herne in Germany. Malnutrition was defined as Mini Nutritional Assessment Long Form (MNA-LF) score of less than 17 [9] or weight loss >10% of initial bodyweight in six months or shorter [10]. Exclusion criteria were age <65 years, missing or withdrawn consent, reduced cognitive abilities (Montreal Cognitive Assessment (MoCA) score of less than 10), severe dementia, severe depression, dysphagia, edema and artificial nutrition.

Bodyweight and REE were measured within 24 h after hospital admission and at the time of discharge. In addition, the geriatric assessment was performed within 24 h after hospital admission. Serum concentrations of thyroid-stimulating hormone (TSH) and C-reactive protein (CRP) were measured on admission according to standard clinical procedures. CRP level ≥2 (mg/dL) was defined as moderate inflammation. The degree of weight loss was obtained by interview or derived from the patients’ medical records. Body composition analysis was performed during the hospital stay. The study protocol had been approved by the ethical committee of Ruhr-University Bochum (17-6217, approved on 18.01.2018). Written informed consent was obtained from all patients.

### 2.1. Nutritional Treatment

All malnourished patients received individualized nutritional therapy, i.e., nutritional counseling and high protein and/or high calorie oral nutritional supplements (ONS) during hospitalization. However, the composition and amount were different based on the patient’s nutritional needs and preferences.

### 2.2. Geriatric Assessment and Body Composition

Activities of daily living were determined using Barthel-Index [11]. FRAIL scale [12] and SARC-F questionnaire [13] were used to identify persons at risk of frailty and sarcopenia, respectively. MoCA [14] was used to evaluate cognitive function, and depressive symptoms were diagnosed using Depression in Old Age Scale (DIA-S) [15]. The Parker mobility score [16] was performed to evaluate the patient’s mobility indoors and outdoors. In addition, FFM, fat mass and (FM), skeletal muscle mass (SMM) were measured using the phase-sensitive, multi-frequency 8 electrode SECA medical Body Composition Analyser 525 device (BIA, seca mBCA 525, Hamburg, Germany).

### 2.3. REE Measured and Predicted

REE_measured_. REE was measured continuously for a minimum of ten minutes by indirect calorimetry (Q-NRG, Cosmed, Rome, Italy) using a clear plastic canopy hood. The first three minutes of every test were discarded. The device was warmed up before each measurement and flowmeter was calibrated using 3 L calibration syringe weekly. In addition, the device was calibrated with a gas mixture of 16% O_2_, 5% CO_2_ and balance nitrogen monthly, as recommended by the manufacturer. The measurement of REE was taken in the morning after an overnight fast >8 h under standardized conditions. REE was calculated from whole-body oxygen uptake and whole-body carbon dioxide production according to Weir equation [17].

REE_predicted_. REE was predicted by using the anthropometric based formula of Harris–Benedict equation [8] as follow:

For females: 655.1 + (9.563 × weight in kg) + (1.850 × height in cm) − (4.676 × age in years)(1)

For males: 66.5 + (13.752 × weight in kg) + (5.003 × height in cm) − (6.755 × age in years).(2)

### 2.4. Data Analysis

Statistical analyses were carried out with SPSS statistical software Version 26.0, (SPSS Statistics for Windows, IBM Corp, Armonk, NY, USA). Continuous variables are reported by means and standard deviations (SDs) for normally distributed variables and median values with interquartile ranges (IQR) for non-normally distributed data. Categorical variables are reported as absolute numbers and percentages (*n*, %). For normally distributed variables, differences in variables on admission and at discharge were tested using an unpaired *t*-test, whereas differences in variables over time were analyzed by using paired samples *t*-test. Categorical variables were compared by the Chi square test. Pearson’s correlation coefficient was performed for relations between variables. The Bland-Altman analysis [18] was used to assess the bias and limits of agreement between REE_measured_ by IC and REE_predicted_ by the Harris–Benedict equation. REE_predicted_ within ±10% of the REE_measured_ was considered as accuracy. In addition, a stepwise multiple regression analysis was used to investigate the effects of different variables on the difference between REE_measured_ and REE_predicted_ (as dependent variables) on admission. These independent variables were—mobility (parker mobility score), FFM, TSH, inflammation (CRP), and malnutrition (MNA-LF score). In order to avoid any auto-correlations, age, gender and weight, which are included in the Harris–Benedict equation, were excluded from the regression analysis. A *p*-value of <0.05 was considered as the limit of significance.

## 3. Results

Table 1 shows the baseline characteristics of study participants. Out of 23 patients, 15 were women. All patients were malnourished with a median MNA-LF score of 14 and mean age 81.8 ± 8.1 years. They were free of either edema or fever. Major diagnoses defined as the reason for hospital admission were cardiovascular diseases, falls, fractures, osteoarthritis and primary neurodegenerative diseases. All patients had probable sarcopenia according to SARC-F and displayed reduced mobility based on the parker mobility score. The majority of the patients were frail (91%) and demonstrated an impaired cognitive function (94%). In addition, 48% of the participants exhibited a severe depressive symptom. Almost half of the population (49.5%, *n* = 11) had moderate inflammation. Nutritional support (i.e., high protein and/or high calorie ONS) was provided to all malnourished patients during hospital stay, although two patients (9%) did not take it at all. Concerning gender, females and males were generally comparable, although height, FFM and SMM values were significantly lower in females than males. In addition, there were no significant differences in REE_measured_ (Males: 1000.7 ± 366.1 (kcal/d) vs. Females: 949.7 ± 195.4 (kcal/d); *p* = 0.722) and REE_predicted_ (Males: 1264.1 ± 180.0 (kcal/d) vs. Females: 1130.1 ± 119.9 (kcal/d); *p* = 0.150) between gender.

REE_measured_ and REE_predicted_ on admission and at the time of discharge are summarized in Table 2. The median time between admission to discharge for REE measurements by IC was 13 days (IQR: 11–15). Both REE_measured_ and REE_predicted_ increased significantly during nutritional therapy. However, the magnitude of changes was more pronounced in REE_measured_ than REE_predicted_ (212.6 kcal vs. 19.5 kcal; *p* = 0.018). REE_predicted_ was significantly higher than REE_measured_ (*p* < 0.001) on admission. This difference disappeared at discharge (*p* = 0.713, Table 2). In addition, the magnitude of deviations of REE_measured_ from the REE_predicted_ was greater on admission than discharge (−223.0 kcal/d vs. −29.8 kcal/d, *p* = 0.018). Indeed, the average REE_predicted_ exceeded the REE_measured_ on admission and at the time of discharge by 29% and 11%, respectively (Table 2).

**Figure 1:**
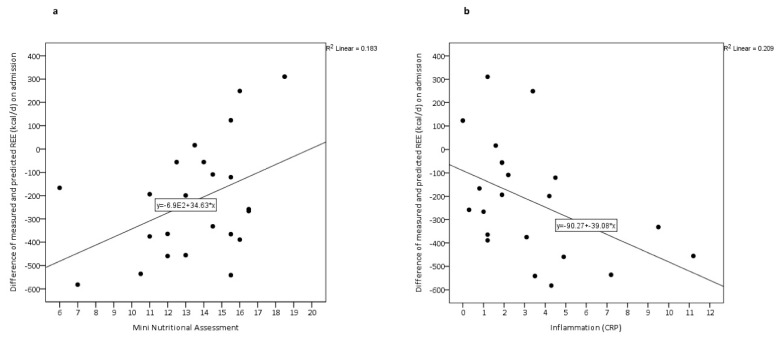
Associations between differences of measured and predicted resting energy expenditure (REE) on admission and (**a**) Mini Nutritional Assessment Long Form and (**b**) inflammation (CRP, C-reactive protein). The solid line is the regression line. REE was measured by indirect calorimetry and predicted by the Harris–Benedict equation.

The differences in REE_measured_−REE_predicted_ on admission were plotted against MNA-LF scores (Figure 1a) and CRP levels (Figure 1b). The magnitude of difference between REE_measured_ and REE_predicted_ was significantly higher in patients with higher CRP levels (r = −0.45, *p* = 0.033; Figure 1b) and increased along with the degree of malnutrition *(r* = 0.42, *p* = 0.042; Figure 1a).

**Figure 2:**
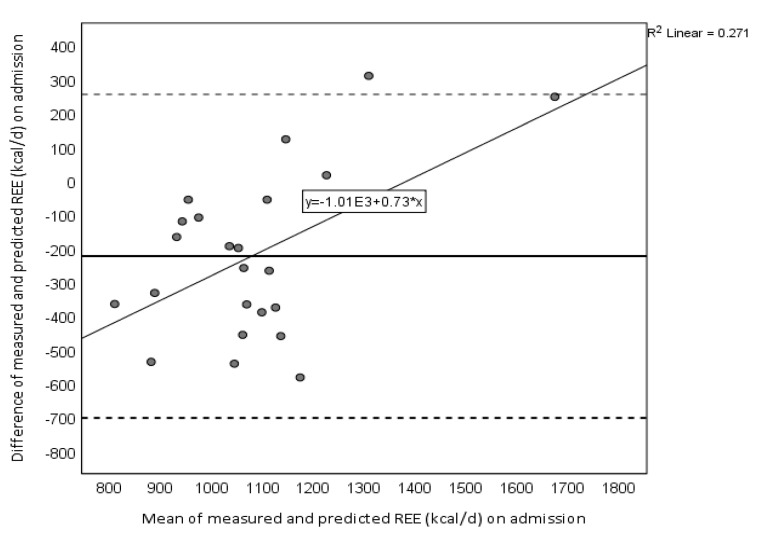
Bland-Altman plots displaying the agreement between measured REE by indirect calorimetry and predicted REE values by the Harris–Benedict equation on admission. Solid line indicates the mean difference and dashed lines indicate ±2 s.d.

In a Bland-Altman plot (Figure 2), changes in REE_measured_−REE_predicted_ correlated with changes in the mean of REE_measured_−REE_predicted_ (*r* = 0.521, *p* = 0.011), which suggests a systematic bias (i.e., the predicted overestimated REE_measured_ at low, but led to an underestimation at high REE). Indeed, REE_predicted_ overestimated the REE_measured_ in 74% of the patients by 41% and underestimated the REE_measured_ in 13% of the patients by 15%. REE_predicted_ was accurate in 13% of the subjects.

There were no significant associations between REE_measured_ and REE_predicted_ on admission (*p* = 0.063) and at discharge (*p* = 0.189). Significant correlation was observed between REE_measured_ and FFM (*p* = 0.043) on admission. In addition, REE_predicted_ was closely related to FM (*p* = 0.028), FFM (*p* = 0.006) and SMM (*p* = 0.002) on admission.

In a stepwise multiple regression analysis, the effects of mobility (parker mobility score), FFM, TSH, inflammation (CRP) and malnutrition (MNA-LF score) (as independent variables) on the difference between REE_measured_ and REE_predicted_ (as dependent variable) on admission were tested (Table 3). Inflammation and MNA-LF score entered the prediction model accounting for 36% of the variance in the deviation between REE_measured_ and REE_predicted_.

## 4. Discussion

The results of this study demonstrated that REE_predicted_ by the Harris–Benedict formula in malnourished older patients deviates 29% from REE_measured_ by IC on admission. In fact, this phenomenon is increasingly apparent with the degree of malnutrition as deviations ranged from −582 to +310 kcal/day in severe to mildly malnourished patients, respectively.

A number of equations have been proposed to predict REE. However, most of the accepted predictive equations are based on healthy not hospitalized patients and equations specifically based on geriatric patients are rather scarce. A review focusing on frail elderly people reported that the Harris–Benedict formula and WHO formulae accurately predicted REE [19]. In another study of 119 healthy older subjects (aged 70–98 years), Melzer et al. [20] showed that the Harris–Benedict formula had 72% accurate predictions. In a literature search, however, few validation studies have investigated malnourished older hospitalized patients and the role of nutritional status in the accuracy and validity of the REE_predicted_ has been paid less attention.

The major finding of the present study is that REE_predicted_ by the Harris–Benedict formula appears to be subject to large errors in malnourished older hospitalized patients on admission. We found that Harris–Benedict formula overestimated REE by 223 kcal/day with high variability between subjects (range 109–582 kcal). Indeed, the ability of Harris–Benedict formula to predict REE was affected by the degree of malnutrition such that the mean largest difference of predicted and measured REE was seen among patients with severe malnutrition (444 kcals, 61%). However, the literature shows that the general variation between both methods might be natural and quite acceptable (REE predicted within ±10% of measured REE) [5,10,21]. Our data demonstrated that in 74% of the patients, REE_predicted_ exceeded the REE_measured_ by 41% and the predicted value was within 10% of the measured value in only 13% of the subjects suggesting that malnourished patients are difficult to predict accurately. In accordance with our results, the findings of a prospective trial in 14 hospitalized patients (mean age 66.5 years) who were severely underweight (bodyweight below 50 kg) demonstrated that the percentage difference between REE measured and REE predicted by the Harris–Benedict formula was 18.4 ± 9.4% [22]. Furthermore, Roza and Shizgal [23] investigated 33 normally nourished patients and 41 malnourished patients and indicated that the Harris–Benedict formula is unreliable in estimating REE in the malnourished group. In another study among 61 hospitalized elderly African-American (mean age 79.6 ± 8.9 years), Compher et al. [24] found that the Harris–Benedict equation significantly underestimated the measured REE. It is also worth noting that in the current study, the mean REE_predicted_ by the HB in males and females were comparable to the results of the HB equation in the 1935 study [25] (Males: 1264.1 ± 180.0 (kcal/d) vs. 1293.0 ± 280.0 (kcal/d), respectively; Females: 1130.1 ± 119.9 (kcal/d) vs. 1108.0 ± 179.0 (kcal/d), respectively).

Moreover, the results of the regression analysis in our study showed that inflammation and malnutrition were the major risk factors in the deviation between REE_measured_ and REE_predicted_ on admission. However, after administration of almost two weeks of individualized nutritional therapy, the percentage difference between REE_measured_ and REE_predicted_ was marginal (11%). In a study of 194 malnourished older hospitalized patients (mean age 74.3 years), the best prediction equations showed 40% accuracy; and three months after discharge, 66% accuracy could be reached [10]. The authors determined that the difference in accuracy of REE between admission and three months after discharge appears to be due the improved health of the patients [10]. The same is true in the present study, since bodyweight (on admission 62.4 ± 11.4 kg, at discharge 64.1 ± 12.1 kg; *p* = 0.097) and health status of the patients improved during 2-weeks nutritional therapy. Nevertheless, the limits of agreement between both methods were still displayed a high variability between subjects, possibly because some of the patients were still in suboptimal health and malnourished. Unfortunately, neither Harris–Benedict formula nor the other accepted predictive equations consider other potentially important variables besides age, gender and weight, such as malnutrition and inflammation, factors that seem to affect the metabolic state. Considering these findings, it seems that the Harris–Benedict equation displays a better accuracy in healthy, non-malnourished older adults and has acceptable accuracy in group comparison rather than in individuals, due to high variability between subjects.

It is of interest to note that, mean REE measurement of 967 kcal/day for malnourished patients in our study was relatively low compared to the mean measured REE among healthy older individuals reported by Geisler et al. (1194 kcal/day) [26], Bosy-Westphal (1368 kcal/d) [27] and Melzer et al. (1370 kcal/d) [20]. Although, IC considered the gold standard for determining appropriate nutritional therapy and reflects the true measurement of energy needs in healthy individuals, we believe that malnutrition decreases measured REE as an energy-saving component of metabolic adaptation. Therefore, measured REE by IC may not represent the patient’s true energy requirements to recover from malnutrition. This finding has important clinical implications as it suggests that relying on REE data obtained by IC may be inadequate to cover all the energy needs in malnourished patients, and therefore, nutritional therapy should be adapted to the individual needs of each patient. Given that, the effect of malnutrition on measured REE must be taken into account when estimating energy needs in these patients.

Some limitations of the study need to be addressed. Body composition was performed by BIA, which is highly influenced by hydration status [28]. That is why we excluded subjects with edema. We did not measure nutritional intake, which is difficult to perform in a geriatric population. In addition, our study has a small sample size mainly due to difficulty in using indirect calorimetry in some older patients, i.e., patients’ fear to use the ventilated canopy hood or patients’ discontent to assess REE two times. However, using the IC, which is considered as gold standard allowed to determine energy requirements precisely in our patients on admission and at the time of discharge.

## 5. Conclusions

REE predicted by the Harris–Benedict formula is not accurate in malnourished older hospitalized patients. However, REE measured by indirect calorimetry is considered precise, but it may not represent the true energy requirements to recover from malnutrition. Therefore, the effect of malnutrition on measured REE must be taken into account when estimating energy needs in these patients.

## Figures and Tables

**Table 1:** Characteristic of the study population on admission.

	All (*n* = 23)
Gender (number, %)	
Females	15 (65)
Males	8 (35)
Age (y)	81.8 ± 8.1
Height (m)	1.63 ± 0.1
Bodyweight (kg)	62.4 ± 11.4
BMI (kg/m^2^)	23.4 ± 4.0
Geriatric assessments, Median (IQR)	
MNA-LF	14 (12–15)
Barthel-Index	45 (40–55)
Parker mobility score	2 (2–4)
Frail Simple score	5 (4–5)
SARC-F scores	8 (6–9)
Depression score (DIA-S)	3 (2–6)
Cognitive function (MoCA)	17 (15–21)
Bioelectrical impedance analysis (kg)	
FM	18.8 ± 9.6
FFM	46.1 ± 7.7
SMM	17.9 ± 4.9
CRP (mg/dL)	3.2 ± 2.9
TSH (mU/mL)	2.1 ± 1.9

MNA-LF, Mini Nutritional Assessment Long Form (normal nutritional status 24–30 points, at risk of malnutrition 17–23.5 points and malnourished <17 points); Parker mobility (ranges 0–9 with a highest overall score of 9 demonstrates the best possible mobility); Frail Simple scale (not frail with score 0, pre-frail with scores of 1–2 and frail with 3–5); SARC-F scores (high risk of sarcopenia with score ≥4); DIA-S scores, Depression in Old Age Scale (no depressive symptom with 0–2 points, suspected depression 3 point and probable depression 4–10 points); MOCA, Montreal Cognitive Assessment (scores <26 considered as cognitively impaired); FFM, fat free mass; FM, fat mass; SMM, skeletal muscle mass; CRP, C-reactive protein; TSH, Thyroid-stimulating hormone. Values are given as a number (%), mean ± SD or median (IQR, interquartile range).

**Table 2:** Measured and predicted REE on admission and at discharge.

	All (*n* = 23)		
	On admission	At discharge	Changes
REE_measured_ (kcal/d)	967.5 ± 260.0	1180.1 ± 397.9	212.6 ± 363.0 ^aa^
REE_predicted_ (kcal/d)	1190.4 ± 152.3 ^b^	1209.9 ± 150.0	19.5 ± 45.7 ^a,b^
REE_measured−_REE_predicted_ (kcal/d)	−223.0 ± 244.2	−29.8 ± 383.3	193.1 ± 360.7 ^aa^
(REE_predicted_/REE_measured_) × 100 (%)	129%	111%	18%

REE_measured_, resting energy expenditure measured by indirect calorimetry; REE_predicted_, REE predicted by using the Harris–Benedict equation; ^a^ < 0.05 and ^aa^ *p* < 0.01 Difference between admission and discharge; ^b^ *p* < 0.001 Difference between REE_measured_ and REE_predicted_. Values are given as mean ± SD.

**Table 3:** Stepwise multiple regression analysis of risk factors associated with the difference between REE_measured_ and REE_predicted._

	Beta Coefficient	SE	*p* Value
Difference between REE_measured_ and REE_predicted_			
Parker mobility score on admission	26.44	46.46	0.647
FFM	6.09	9.18	0.231
TSH	−25.03	53.33	0.826
Inflammation (CRP)	−33.88	15.88	0.046
Total MNA-LF	31.96	15.09	0.048

REE_measured_, resting energy expenditure measured by indirect calorimetry; REE_predicted_, REE predicted by the Harris–Benedict equation; FFM, fat free mass; TSH, Thyroid-stimulating hormone; CRP, C-reactive protein; MNA-LF, Mini Nutritional Assessment Long Form; SE, standard error.
